# The Psychosocial Impact of Urinary Dysfunction

**DOI:** 10.5152/tud.2024.23217

**Published:** 2024-05-01

**Authors:** Stephanie Gleicher, Elisabeth M. Sebesta, Roger R. Dmochowski

**Affiliations:** 1 The Smith Institute for Urology, Northwell Health, Lakeville Drive, Lake Success, NY, USA; 2 Department of Urology, Vanderbilt University Medical Center, Nashville, TN, USA

**Keywords:** Psychology, social, urinary symptoms

## Abstract

Urinary dysfunction encompasses a wide range of syndromes and symptoms and is highly prevalent among the adult population. Urinary issues have been associated with psychosocial sequelae. The interplay between psychosocial comorbidity and symptoms impacts perceived severity and treatment success. While the correlation has been described in the literature, much remains unknown. This article describes the psychosocial impact on conditions such as overactive bladder (OAB), neurogenic lower urinary tract dysfunction (LUTD), recurrent urinary tract infection (UTI), and interstitial cystitis/bladder pain syndrome (IC/BPS). This article also highlights potential interventions for patients afflicted with both urinary disorders and psychosocial comorbidity to improve overall treatment success.

## Introduction

Nearly three quarters of adults are afflicted with urinary dysfunction, which encompasses a wide range of urinary symptoms and syndromes.^1^ Lower urinary tract symptoms (LUTS) include storage, voiding, and post-micturition symptoms encompassing nocturia, urinary urgency, urinary frequency, incontinence, incomplete emptying, stream changes, and post-void dribble.^2^ Syndromes, such as overactive bladder (OAB), encompass several storage symptoms and have been linked to significant psychological sequelae.^3-5^ Other urinary dysfunction subtypes include recurrent urinary tract infections (rUTIs), neurogenic lower urinary tract dysfunction (NLUTD), and interstitial cystitis/bladder pain syndrome (IC/BPS). Recurrent urinary tract infection is defined as 2 or more UTIs in 6 months or 3 or more UTIs in a 12-month period.^6^ Although less well studied, literature has suggested increased anxiety, worse health perception, poorer perceived quality of life (QOL), and increased functional impairment among subjects with rUTI.^7^ Neurogenic lower urinary tract dysfunction describes a constellation of urinary symptoms and chronic bladder conditions that result from neurologic conditions of both the central and peripheral nervous systems, both acquired and congenital. It refers to the “abnormal function of either the bladder, bladder neck, and/or sphincters” related to a known neurologic disorder.^8^ Neurogenic lower urinary tract dysfunction significantly affects psychosocial domains, with significant psychosocial burden experienced by both those with NLUTD and family members, friends, and/or caregivers.^9^ Lastly, IC/BPS is defined by symptoms including pelvic pain, urinary urgency, bladder pressure or discomfort, and urinary frequency.^10^ The symptoms of IC/BPS have huge impacts on quality of life (QOL) and can be highly debilitating to both physical and emotional functioning. The condition is considered a biopsychosocial disorder, where biological and psychosocial factors intersect to produce the symptoms and experience of IC/BPS.^11^

As more research is published in this realm, the association and correlation between urinary issues and psychosocial sequela cannot be ignored.^3,5,12^ For healthcare providers who manage patients suffering from these forms of urinary dysfunction, psychosocial comorbidities should be considered in patient treatment algorithms. The aim of this report is to summarize the psychosocial impact of various urinary conditions and offer suggestions for management. To accomplish this goal, a review of recent literature was performed regarding the psychosocial association with OAB, NLUTD, IC/BPS, and rUTI as well as possible interventions. We also described various psychosocial scales/domains commonly implemented to assess psychosocial comorbidity as well as the concept of coping.

## Assessment of Psychosocial Domains

It is important to understand the construct of validated scales that are commonly utilized to systematically screen and classify psychosocial sequelae and comorbidities. One scale is the Short Form 36 health survey questionnaire (SF-36). The SF-36 addresses functional status, wellbeing, and overall health.^13^ Within each area, questions are used to assess physical and social functioning, physical and emotional problems, mental health, energy, pain, and overall health perception.^13^ Scoring is based on a scale of 0 to 100 (best health). Abbreviated versions of the SF-36 have been devised, including the SF-12, which correlates highly with the SF-36 (*R*-scores > 0.9 for physical and mental health parameters).^14^

The Hospital Anxiety and Depression Scale (HADS) is a brief outpatient screening tool for anxiety and depression.^15,16^ Comprised of 14 questions, scores range from 0- 21 with higher scores indicative of a higher likelihood of psychiatric comorbidities. Scores between 0 and 7 imply no mental health issue, while scores 8-10 are considered “doubtful.” Scores between 11 and 21 have a stronger correlation with a true diagnosis of anxiety and depression.^16^

The Leicester Impact Scale was designed to assess the impact of urinary storage symptoms on activities (i.e., shopping, visiting people, social and family life, trips, overnight stays, and hobbies) and feelings (i.e., anger, depression or anxiety, embarrassment, frustration, worry, shame, guilt, perceived attractiveness).^17^ Each subscale of this 21-question survey is tallied, and a total composite score is generated.^17^

The Patient Reported Outcomes Measurement Information System (PROMIS®) is an NIH-funded validated scale used to characterize physical, mental, and social health parameters.^18^ Emotional distress correlates with anxiety, depression, and anger. Short forms were designed with 7 and 8 questions regarding anxiety and depression, respectively.^18^ The PROMIS Assessment Center Scoring Service calibrates responses and produces a standardized *T*-score, which is compared to the general population.^19^

The Perceived Stress Scale (PSS) is a validated survey that assesses how subjects deem their life to be unpredictable, uncontrollable, and overloaded.^20^ Surveys are scored, with higher scores correlating with higher perceived stress. The original PSS included 14 items, but short forms with 4 and 10 items have also been validated.^20^

## Psychosocial Impact on Coping

A concept that emerges when exploring psychosocial sequela in urinary conditions is coping. Coping is a dynamic response to both external and internal stress that allows for adaptability and self-regulation.^21,22^ Coping strategies are further subdivided into primary and secondary control. Primary control includes both problem-based techniques (i.e., diminish the stressor, problem solving) and emotion-based strategies (i.e., regulate emotions, avoidance).^22^ Secondary control refers to cognitive restructuring or acceptance of circumstances.^22^ Generally, problem-based strategies have been shown to lessen psychological burden, while emotion-based have the potential to worsen adjustment to and potentiate symptoms of illness.^23,24^ More recent literature has elucidated various coping mechanisms among individuals suffering from urinary dysfunction^24,25^

## Psychosocial Impact in OAB

Overactive bladder is a complex of symptoms including urinary urgency with or without incontinence (OAB wet versus OAB dry), as well as urinary frequency and nocturia.^2^ It is estimated that nearly one-quarter of adults in the United States (US) suffer from OAB.^26^ Adults with OAB have been shown to have worse sleep, more depression, and worsened quality of life (QOL).^1,3,5,12^ Individuals with OAB also have higher rates of absenteeism and impaired functionality.^26^ These individuals have been found to implement more coping strategies.^12^

The epidemiology of lower urinary tract symptoms (EpiLUTS) study highlighted the correlation between various LUTS and psychosocial sequela.^3^ This observational study primarily assessed the prevalence of LUTS among adults from various countries but also assessed the psychosocial impact of these same symptoms. The EpiLUTS study found a significant association between nocturia and urgency with anxiety for both genders, as well as a correlation between urgency and depression among women.^3^ Subjects reporting OAB with bother had the highest rates of anxiety and depression.^27^ The study also showed that significantly fewer individuals with OAB worked full time, and individuals reporting OAB with bother were more likely to report permanent disability.^27^ A systematic review was conducted on the psychosocial burden of OAB, which included 32 studies and addressed the association between OAB with depression, anxiety, embarrassment/shame, self-esteem, sleep, relationships, social life, and QOL.^4^ Patients with OAB were found to have difficulties in each domain, with an even more pronounced association among subjects with OAB wet.^4^ These studies highlight the profound impact of OAB on psychosocial distress.

Lai et al reported significantly higher stress levels among women with OAB compared to the general population.^28^ They found that OAB patients with a history of sexual trauma, higher anxiety scores, and higher depression scores had higher stress.^28^ Lai et al also showed a significant correlation between perceived stress and self-reported urinary symptom severity.^28^ They suggested psychological distress may be contributing to OAB symptom severity, and that stress reduction may improve symptoms.^28^ A recent study showed that women with OAB and underlying anxiety reported a greater psychosocial burden of disease.^29^ Individuals with OAB and concomitant anxiety reported significantly more stress and somatic symptom burden versus subjects with OAB and no anxiety.^29^ While prior reports discuss the increased rates of anxiety among subjects with OAB, this study elucidates how perceived symptom severity may manifest based on concomitant psychosocial distress.^29^

A recent study highlighted compensatory coping strategies to manage OAB symptoms.^24^ The authors showed a correlation between higher emotion-based coping composite scores (based on utilizing more strategies of discomfort traveling, avoiding activities, decreasing exercise, and seeking escape routes) with perceived OAB symptom severity.^24^ They also found that higher coping scores correlated with significantly greater anxiety and perceived stress in multivariate models.^24^ While these behaviors lessen the psychological burden of OAB in the moment, they promote and maintain the psychosocial connection with urinary dysfunction. These behaviors need to be considered when managing OAB symptoms.

## Psychosocial Impact in NLUTD

The psychological comorbidity associated with NLUTD has been reviewed previously, as well as the opportunity for multidisciplinary care of these complex patients.^9^ Urinary function is among the top priorities for functional recovery in patients with spinal cord injury (SCI), on par with the ability to walk following an injury.^30,31^ Therefore the impact of NLUTD on psychosocial health and QOL, specifically, is quite significant.

Previous studies suggest that people with NLUTD experience increased psychological distress. They may exhibit increased social isolation and a reduction in self-esteem, which ultimately may lead to worse long-term health outcomes, poorer rehabilitation post-neurologic injury, and poorer perceptions of their overall health.^32^ Likewise, there is extensive overlap between NLUTD and psychological conditions. Anxiety, including anxiety specifically related to bladder symptoms and conditions, is incredibly common. Bladder-specific anxiety includes anxiety around incontinence episodes, lack of access to adequate bathrooms while in public, and fear of worsening symptoms. This bladder-specific anxiety additionally results in increased social isolation, feelings of shame, embarrassment, or loss of dignity, and poorer self-esteem and self-confidence.^33,34^ Similarly, depression, which often exists comorbidly with anxiety, is quite common in NLUTD.^35^ The prevalence of major depression in patients with SCI ranges from 10 to 40% in the existing literature.^36^ Depression has been associated with increased urinary symptom severity in people with NLUTD, which is likely a complex and bidirectional relationship.^4,9^ Increased psychosocial burden, whether depression, anxiety, psychological distress, or other, has been associated broadly with poorer overall health outcomes.^3,9,36^ This includes increased hospitalizations, an increased incidence of substance use disorders, decreased functional status, decreased overall life expectancy, and an increase in all-cause mortality, including increased suicide rates.^9,36,37^

Similarly, the impact of NLUTD on interpersonal relationships and social supports is quite complex. As stated above, social relationships are vastly impacted by a patient’s perceived embarrassment or shame surrounding their urinary management and bladder function—including incontinence, catheters, and urinary symptoms, the perception of loss of control, and the feeling of needing to plan social events around their bladder or urinary management.^38^ The increased psychological burden associated with NLUTD can increase social isolation and disengagement, so much so that the less severe the urinary dysfunction in a person with NLUTD, actually the less social isolation and more social engagement that occurs.^39^ There is some evidence that good urologic management of NLUTD, which aims at increased autonomy, can increase confidence and thereby lead to social re-engagement.^40,41^

Other interpersonal relationships are also affected, including sexual and intimate partnerships and caregiver relationships. Not only is there concomitant sexual dysfunction due to underlying neurologic disease, but urologic management of NLUTD can impact sexual relationships as well. Incontinence during sexual activity is a frequently cited source of anxiety, resulting in embarrassment and the desire to isolate or fear a lack of understanding by sexual partners.^40^ Likewise caregiver relationships in NLUTD may be affected. Specifically, there is the concept of “role changing” that occurs when one has acquired NLUTD, resulting in a loss of independence.^40^ Family and friends may suddenly assume caregiver roles whereas they would not normally be involved in urologic care, and this can strain the relationship for both parties.

Neurogenic lower urinary tract dysfunction can result in profound impacts on psychological health, psychosocial burden, interpersonal relationships, and social engagement. Understanding how urologic care may impact these factors is crucial when considering management strategies with these complex patients.

## Psychosocial Impact of rUTI

Women suffering from rUTI have been shown to have higher rates of mental health comorbidities.^42,43^ Naber et al reported that women with rUTI had worsened general health perception, energy levels, and social and emotional function.^7^

Women with rUTI have higher baseline anxiety, which is believed to be related to a fear of the acute onset of symptoms.^42^ Wagenlehner et al showed that women with an acute UTI, versus women who had a UTI in the past 4 weeks, had worse physical function scores but persistently low mental health scores despite the resolution of infection.^43^ Renard et al enrolled adult women with rUTI in a 6-month study to determine the impact of an rUTI prophylaxis regimen and fewer UTI episodes.^42^ Ultimately, women incurred a 59% reduction in UTIs, which correlated with a significant improvement in mental health; rates of clinical anxiety decreased by 36%, and rates of clinical depression decreased by 25%.^42^ With rUTI prevention protocols, the overall Leicester impact score on activities and feelings decreased by 33% and 55%, respectively.^42^

Compared to adult women who had an antibiotic prescribed for a UTI within 60 days, subjects with rUTI reported significantly higher rates of an impaired ability to carry out household chores, partake in social activities, and run errands.^44^ In general, women who incurred and were treated for UTIs in the prior 2 months reported worse QOL and overall work impairment versus control-matched populations.^44^ Grigoryan et al conducted interviews with adult women who had at least one treated UTI in the past year in both the US and Germany.^45^ Primary themes included feelings of “frustration, hopelessness, and loss of control” regarding daily activities.^45^ Women also felt that UTIs made them feel isolated and embarrassed regarding relationships.^45^ Cost burden also contributed to feelings of stress, with subjects reporting “worry about being able to cover other living costs”.^45^

## Psychosocial Impact of IC/BPS

The psychosocial burden and psychological comorbidity of IC/BPS may substantially affect other aspects of a person’s life, including social relationships, daily activities, intimate and sexual relationships, and work productivity or employment stability.

There has been increasing research over the last decade on psychosocial factors and chronic pain conditions, including chronic urologic and pelvic pain, particularly with the formation of the Multidisciplinary Approach to the Study of Chronic Pelvic Pain (MAPP) network.^46^ A recent systematic review on the psychological comorbidity of IC/BPS found that depression occurs in as many as 70% of cases, and anxiety ranges between 14% and 52%.^47^ Those with IC/BPS were found to be 5 times more likely to be diagnosed with a pre-existing anxiety disorder. Other common psychosocial comorbidities include low self-esteem, catastrophizing, generalized stress, and trauma. Post-traumatic stress disorder (PTSD) was found in 42% of those with IC/BPS in a recent study, which was significantly higher than the incidence of PTSD in those with general chronic pain. Those with PTSD had significantly increased rates of prior abuse and childhood trauma and experienced worse health outcomes, including QOL, pain severity, and increased emotional distress.^11^ There is an increased incidence of both suicidal ideation and suicide attempts in people with IC/BPS, with recent studies demonstrating a high prevalence of suicidal risk (38%), and increased recent suicidal ideation as compared to healthy controls.^48,49^ Lastly, more than 70% of individuals with IC/BPS endorse emotion-based coping skills (i.e., avoiding activities away from the restroom), which reinforce the urinary symptoms and the experience of pain.^25^ This learned compensatory coping has the potential to undermine the resolution of symptom burden. Central sensitization, which is an abnormal processing of stimuli as pain, is one explanatory model for the psychosocial and pathophysiologic changes of IC/BPS. In sensitization, normally mild stimuli may produce increased sensation, increasing pain intensity. It is thought that psychological and physical stress systems additionally interact, such that once neutral stimuli now provoke a threat response that is not only physical (painful) but psychological (anxiety, distress, etc.).^50,51^ Understanding IC/BPS as a biopsychosocial disease is crucial in offering the interdisciplinary care needed by these patients.

## Potential Intervention for Psychosocial Burden

There is a paucity of literature on interventions to address the psychological comorbidity and psychosocial burden associated with LUTS and bladder conditions. While we know that cognitive behavioral approaches can be beneficial in addressing coping mechanisms, stressors, and psychological comorbidity, the actual effects of these types of interventions on urologic symptoms are not well documented.

Cognitive behavioral therapy (CBT) is a form of psychological treatment that has been used in a wide range of conditions. It combines cognitive and behavioral approaches to address interrelated thoughts, feelings, perceptions, physical sensations, and actions. The idea behind CBT is to gain insight into distorted attitudes and perceptions and coping behaviors that are dysfunctional or maladaptive, which create a reinforcing cycle. By addressing these attitudes and thinking patterns and the learned patterns of behavior and coping, CBT results in improvements in QOL and overall functioning. With regards to IC/BPS specifically, there is increasing evidence to highlight the need for psychosocial interventions as a part of overall treatment. The need for multidisciplinary integrative care for these patients is clearly recognized, particularly with the need for stress management and coping, due to the heightened pain sensitivity that is associated with psychological stress.^52^ Although limited, studies suggest that implementing psychosocial interventions can result in decreased pain, improvement in symptoms, and overall improvement in QOL. However, research in this space is still needed.

In terms of urologic symptoms and bladder conditions, there is some evidence to suggest CBT may be helpful. Cognitive behavioral therapy may be useful in addressing the negative self-perception and self-esteem that comes with urinary symptoms. A recent randomized clinical trial on women with incontinence demonstrated that self-esteem and sexual function improved significantly after 8 CBT sessions, which was maintained a month after completing the sessions.^53^ A recent systematic review examined the use of CBT in UUI, either as a standalone therapy or in conjunction with pelvic floor muscle training (PFMT).^54^ Although the studies included were quite heterogeneous, the authors concluded, based on 12 available studies, that CBT with or without PFMT resulted in improvements in UUI severity, in addition to improvements in QOL, patient satisfaction, and psychological symptoms, as compared to no treatment, and that CBT should be considered as first-line therapy for OAB/UUI.

Clinical hypnosis is a psychosocial intervention in which a trained provider guides a patient into a focused and relaxed state and then suggests changes in sensations, thoughts, emotions, and behaviors.^55^ It is also possible for patients to learn to self-hypnotize as a coping skill. While there is data on the use of hypnosis in the pain space, there is limited information on its implementation in urinary symptom management. A recent pilot study on people with chronic pain and concurrent LUTS found significant improvement in LUTS with the use of group clinical hypnosis over a 6-month period, which was most significant for those with moderate or severe LUTS at baseline.^56^ This improvement in symptoms was independent of any pain improvement seen. Hypnosis has also been applied specifically to women with OAB and UUI, and in a randomized trial, hypnosis demonstrated benefits non-inferior to oral medications after 12 months.^57^ Interestingly, those on medications did respond more quickly, but after 6-12 months the benefits were equal between groups, suggesting improvements with hypnotherapy may increase over time.

Alternative therapies, including yoga, meditation, and mindfulness, have also been investigated in chronic diseases with varying success, including in the management of urinary symptoms. In a small case series on the use of integrated yoga, patients with multiple sclerosis demonstrated improvements in post-void residual volumes and incontinence after 3 weeks of therapy.^58^ Likewise, in a larger series on the use of an integrated yoga practice, including yoga postures, breathing exercises, sound therapy, and meditation in people with incontinence, there were significant improvements in the severity of incontinence and in incontinence-specific QOL after 8 weeks of therapy.^59^ Finally, a small pilot study on mindfulness-based stress reduction in the treatment of UUI in women demonstrated its effectiveness with a decrease in incontinence episodes per day and improvements in QOL after 8 weeks.^60^ While limited literature exists in this space, there is some evidence to suggest the feasibility of such treatments.

Data on psychosocial interventions for the treatment of LUTS is very limited; however, the small amount of data suggests such alternative interventions may serve as important adjuncts in patient care to improve urinary symptoms and QOL. Further research on the use of these techniques is important to determine which patients will benefit the most.

## Conclusion

Urinary dysfunction has a strong association with psychosocial comorbidity, which may influence symptom perception and the successful treatment of patients. It is important to recognize and counsel patients about the impact of various urinary issues on emotional health. While much remains unknown and psychosocial treatment remains outside the management guidelines for these urinary conditions, providers might consider integrative approaches to improve patient outcomes.
